# Effect of mountain ultra-marathon running on plasma angiopoietin-like protein 4 and lipid profile in healthy trained men

**DOI:** 10.1007/s00421-019-04256-w

**Published:** 2019-11-09

**Authors:** Monika Górecka, Krzysztof Krzemiński, Monika Buraczewska, Agnieszka Kozacz, Jan Dąbrowski, Andrzej Wojciech Ziemba

**Affiliations:** Department of Applied Physiology, Mossakowski Medical Research Centre, Polish Academy of Sciences, 5 Pawińskiego str., 02-106 Warsaw, Poland

**Keywords:** ANGPTL4, Ultra-marathon, Triacylglycerols, Free fatty acids, Cholesterol

## Abstract

**Purpose:**

Angiopoietin-like protein 4 (ANGPTL4) regulates lipid metabolism by inhibiting lipoprotein lipase activity and stimulating lipolysis in adipose tissue. The aim of this study was to find out whether the mountain ultra-marathon running influences plasma ANGPTL4 and whether it is related to plasma lipid changes.

**Methods:**

Ten healthy men (age 31 ± 1.1 years) completed a 100-km ultra-marathon running. Plasma ANGPTL4, free fatty acids (FFA), triacylglycerols (TG), glycerol (Gly), total cholesterol (TC), low (LDL-C) and high (HDL-C) density lipoprotein-cholesterol were determined before, immediately after the run and after 90 min of recovery.

**Results:**

Plasma ANGPTL4 increased during exercise from 68.0 ± 16.5 to 101.2 ± 18.1 ng/ml (*p* < 0.001). This was accompanied by significant increases in plasma FFA, Gly, HDL-C and decreases in plasma TG concentrations (*p* < 0.01). After 90 min of recovery, plasma ANGPTL4 and TG did not differ significantly from the exercise values, while plasma FFA, Gly, TC and HDL-C were significantly lower than immediately after the run.

TC/HDL-C and TG/HDL-C molar ratios were significantly reduced. The exercise-induced changes in plasma ANGPTL4 correlated positively with those of FFA (*r* = 0.73; *p* < 0.02), and HDL-C (*r* = 0.69; *p* < 0.05). Positive correlation was found also between plasma ANGPTL4 and FFA concentrations after 90 min of recovery (*r* = 0.77; *p* < 0.01).

**Conclusions:**

The present data suggest that increase in plasma FFA during mountain ultra-marathon run may be involved in plasma ANGPTL4 release and that increase in ANGPTL4 secretion may be a compensatory mechanism against fatty acid-induced oxidative stress. Increase in plasma HDL-C observed immediately after the run may be due to the protective effect of ANGPTL4 on HDL.

## Introduction

Long-distance running, such as marathon or ultra-marathon, is a popular type of physical activity that improves the metabolism of carbohydrates, fats and proteins, thus preventing obesity, diabetes and cardiovascular diseases. The average intensity of marathon is 60% of maximal oxygen uptake (*V*O_2max_) in slow runners, 70–75% in fast runners and 86% in elite runners (Sjödin and Svedenhag 1985; O’Brien et al. 1993). During ultra-marathon, the average intensity is around 50–55% of *V*O_2max_ (Rauch et al. 1998; Neumayr et al. 2003; Helge et al. 2007).

It was estimated that the rate of fatty acid oxidation reaches the maximal values during running at the intensity of 59 ± 3% of *V*O_2max_ (Achten et al. 2003). Utilization of fatty acids as an energy source increases with the prolongation of the exercise (Helge et al. 2007) and it remained elevated during few hours after exercise (Kimber et al. 2003; Henderson et al. 2007). Fatty acids oxidized by skeletal muscles may originate from three sources: lipolysis of endogenous triacyloglycerols (TG) in myocytes (Watt et al. 2002), hydrolysis of TG stored in adipose tissue and hydrolysis of TG contained in lipoproteins such as very low-density lipoproteins (VLDL) and chylomicrons (Helge et al. 2001; Morio et al. 2004). The latter is mediated by the catalytic action of the lipoprotein lipase enzyme (LPL) on the luminal surface of microvascular endothelial cells (Kersten 2014). Baseline activity of the LPL in both skeletal muscle and adipose tissue was found to be greater and the plasma high-density lipoprotein cholesterol (HDL-C) concentration higher in long-distance runners than in untrained subjects (Nikkilä et al. 1978; Vaisberg et al. 2012). Additionally, trained men presented low plasma concentrations of TG, total cholesterol (TC) and low-density lipoprotein-cholesterol (LDL-C) (Hardman 1998; Tomaszewski et al. 2004).

One of the most important factors regulating LPL activity is angiopoietin-like protein 4 (ANGPTL4). This endogenous, circulating inhibitor of LPL is synthesized and secreted by the liver, adipose tissue, skeletal muscle, intestine and heart (Kersten et al. 2009; Staiger et al. 2009; Robciuc et al. 2012; Dijk and Kersten 2014).

It was established that ANGPTL4 expression is stimulated by various molecular mechanisms including peroxisome proliferator-activated receptors (PPAR), glucocorticoid receptor or hypoxia-induced factor α (Staiger et al. 2009; Koliwad et al. 2012; Drager et al. 2013). It was demonstrated that prolonged fasting, caloric restriction, hypoxia, inflammation and cycling are important inducers of plasma ANGPTL4 (Kersten et al. 2009; Tjeerdema et al. 2014; Barker et al. 2016).

ANGPTL4 is a multifunctional protein involved in regulation of lipid and glucose metabolism, energy expenditure, angiogenesis and inflammation (Dijk and Kersten 2014; Tjeerdema et al. 2014; McQueen et al. 2017; Gusarova et al. 2018). Several studies showed that ANGPTL4 inhibits LPL activity at the endothelial cell surface and promotes intracellular degradation of LPL, leading to reduction of the TG-derived fatty acids uptake into tissues (Robciuc et al. 2012; Makoveichuk et al. 2013; Dijk et al. 2016). On the other hand, ANGPTL4 stimulates intracellular adipocyte lipolysis, leading to elevation of plasma free fatty acids and glycerol levels (Mandard et al. 2006; Gray et al. 2012; McQueen et al. 2017). Genetic inactivation of ANGPTL4 (variant E40K) in the human population has been shown to be strongly associated with decreased plasma TG and LDL-C and increased HDL-C concentrations (Romeo et al. 2007).

Human and animals studies showed that exercise markedly induces expression of ANGPTL4 in adipose tissue, liver and skeletal muscles (Catoire et al. 2012, 2014; Norheim et al. 2014; Ingerslev et al. 2017). It was found that both fatty acids and cortisol have the potential to increase ANGPTL4 expression in skeletal muscle during exercise (Catoire et al. 2012, 2014; Norheim et al. 2014). Ingerslev et al. (2017) found that exercise-induced secretion of ANGPTL4 from the liver is driven by a glucagon-cAMP-protein kinase A pathway.

Some studies that investigated the effect of cycling on plasma ANGPTL4 showed that 45 min of cycling at 70% of *V*O_2max_ does not affect plasma ANGPTL4, whereas 120–180 min cycling at 40–50% of *V*O_2max_ increases plasma ANGPTL4 in untrained healthy men (Kersten et al. 2009; Catoire et al. 2014; Norheim et al. 2014). In contrast, in trained healthy men, significant increases in plasma ANGPTL4 were observed immediately after 45-min cycling at 70% of *V*O_2max_ (Norheim et al. 2014). Increases in both plasma ANGPTL4 concentration and skeletal muscle ANGPTL4 mRNA expression were observed also between 60 and 240 min after moderate-intensity exercise in both untrained and trained subjects (Kersten et al. 2009; Catoire et al. 2014; Norheim et al. 2014; Ingerslev et al. 2017; Knuiman et al. 2018).

The aim of this study was to determine whether the 100 km of mountain ultra-marathon running influences plasma ANGPTL4 and whether it is related to plasma lipid changes. The second aim was to find out whether the early post-exercise changes in plasma lipid concentration are related to those of ANGPTL4.

The mountain ultra-marathon was chosen as the stimulus because energy consumption and demand for fatty acids during this type of exercise are much higher than during cycling or running on level ground. It has been shown that the maximum rate of fat oxidation over a wide intensity range are significantly higher during running than in the cycling at the same relative intensities expressed as a percentage of the maximum work load or percent of maximum oxygen uptake (Capostagno and Bosch 2010). This may be due to several physiological and mechanical differences between running and cycling (Chenevière et al. 2010).

## Materials and methods

### Subjects

The study included ten well-trained endurance runners with 5 years of experience with physical training and endurance events. All subjects underwent a medical examination and gave their informed consent to participate in the investigations. The values of serum lipid parameters of the subjects were within normal range. The research procedure was approved by the Local Ethics Committee at Medical University of Warsaw.

The general characteristics of the subjects are presented in Table 1.

**Table 1:** Subject characteristics (the values are mean ± SEM; *n* = 10)

Parameters	
Age (years)	31.0 ± 1.1
Body height (cm)	177.5 ± 1.9
Body mass (kg)	71.5 ± 2.0
Body mass index (kg/m^2^)	22.7 ± 0.4
Maximal oxygen consumption (ml/kg/min)	57.8 ± 2.5
Running history (years)	5.3 ± 1.2
Training volume (km/week)	93.5 ± 3.6
Training time (h/week)	11.0 ± 0.7
Running time in present race (h)	13.4 ± 0.7
Running speed in present race (km/h)	7.6 ± 0.3

### Experimental protocol

The subjects were recruited from ultra-marathoners who completed the mountain ultra-marathon for a distance of 100 km (Krynica Zdroj - Race 7 Valleys). The highest point of the route was at 1262 m a.s.l. and the total altitude gain (i.e., the sum of every gain in elevation throughout an entire run) was 4500 m.

The run was held in September when the air temperature oscillates between 12 and 24 °C with 55–60% humidity. The race started at 4:00 a.m., and the runners had to reach the finish line within 17 h. The running speed was 7.6 ± 0.3 km/h and the subjects reached the finish line within 13.4 ± 0.7 h. The organizer provided a total of seven aid stations (at approximately every 14.5 km) offering an abundant variety of food and beverages in standardized size portions. During the race participants ate and drank high-carbohydrate meals and liquids (sandwiches, cookies, carbohydrate bars, carbohydrate drinks contain 40 g of carbohydrates per serving —500 ml) in the same amount. Ingestion of solid food and fluids was determined according to the reports of the runners using a food calorie table (Kunachowicz et al. 2017). Nutrient analysis revealed a mean intra-race energy intake of 4422 kcal, with 88.3% derived from carbohydrates, 6.8% from fat and 4.9% from protein. Fluid intake varied widely, 8.3–10.2 l, with a mean of 9.3 l.

One week before the run, the subjects were submitted to the incremental, graded exercise test on a treadmill (SensorMedics 2000 Treadmill, USA) until volitional exhaustion to determine their maximal oxygen uptake (*V*O_2max_). The treadmill speed started at 6 km/h and increased by 2 km/h every 3 min. Pulmonary ventilation, O_2_ uptake and CO_2_ production were recorded using the Vmax 29 system (SensorMedics T2000, USA).

On the day of the ultra-marathon, after 30 min of pre-run resting period, immediately after the run and at the 90 min of recovery, blood samples were taken from the antecubital vein for determination of the plasma ANGPTL4, TG, free fatty acids (FFA), Gly, TC, LDL-C, HDL-C concentrations. Blood samples were collected in EDTA containing tubes and centrifuged at 1700×*g* for 15 min at 4 °C. Plasma samples were frozen at − 20 °C and then transported and stored at − 80 °C until assay. Whole-blood samples were used to measure the haematocrit.

The 90-min recovery period was selected based on the data from literature (Kimber et al. 2003; Henderson et al. 2007).

### Blood sample analysis

The plasma ANGPTL4 concentration was measured by enzyme-linked immunosorbent assay using DuoSet ELISA Development kit (R&D Systems, Minneapolis, MN, USA) that recognized full-length ANGPTL4 in human plasma (Smart-Halajko et al. 2010). The intra-assay coefficient of variation of this method was 7.6% ± 0.7 (*n* = 70), while inter-assay coefficient of variation was 16.8% ± 1.8 (*n* = 33). The plasma FFA concentrations were measured using standard enzymatic colorimetric assays (ACS-ACOD Method, Wako Chemicals GmbH, Germany). The intra-assay coefficient of variation was 1.5%. The plasma concentrations of TG, Gly, TC, LDL-C and HDL-C were determined by enzymatic methods using manual diagnostic kits (Randox Laboratories Limited, United Kingdom). The intra-assay coefficients of variation for these assays did not exceed 6.4%. Haematocrit was measured in duplicate. Whole blood samples (~ 9 μl) were transferred to heparinized microcapillary tubes and analyzed by an automated system following microcentrifugation. Haematocrit was used to calculate percent changes in plasma volume. The percentage changes in plasma volume were calculated using Van Beaumont's equation (1972).

### Statistical analysis

The data are presented as means with standard errors (SEM). The normality of variables’ distribution was checked with the Shapiro–Wilk test. The homogeneity of variances was tested with the Levene test. Normally distributed parameters were compared using one-way analysis of variance (ANOVA) for repeated measures. When the ANOVA revealed a statistically significant effect (*p* < 0.05), the Bonferroni test was used for “post hoc” comparisons. Variables with non-normal distribution (FFA, TG, Gly, TG/HDL-C), were compared using the Friedman ANOVA rank test and the Wilcoxon test with the Bonferroni correction for multiple comparisons (*p* < 0.02). In addition, correlation coefficients were calculated between variables using the linear regression analysis, and *p* < 0.05 was accepted as the level of significance. For calculations, the Statistica (2001) version 6. (Statsoft Inc., Tulsa, OK, USA) was used.

## Results

**Fig. 1:**
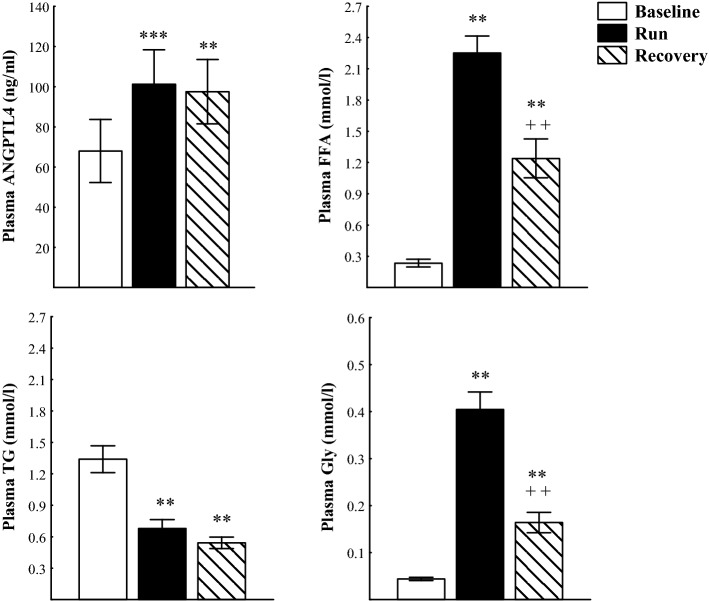
The plasma concentrations of angiopoietin-like protein 4 (ANGPTL4), free fatty acids (FFA), triacylglycerols (TG) and glycerol (Gly) before (Baseline), immediately after ultra-marathon run (Run) and at 90 min of recovery (Recovery). The values are mean ± SEM (*n* = 10). Asterisks denote significant differences from the resting values: **p < 0.01, ***p < 0.001. Crosses denote significant differences from the exercise values: ^++^*p* < 0.01

**Fig. 2:**
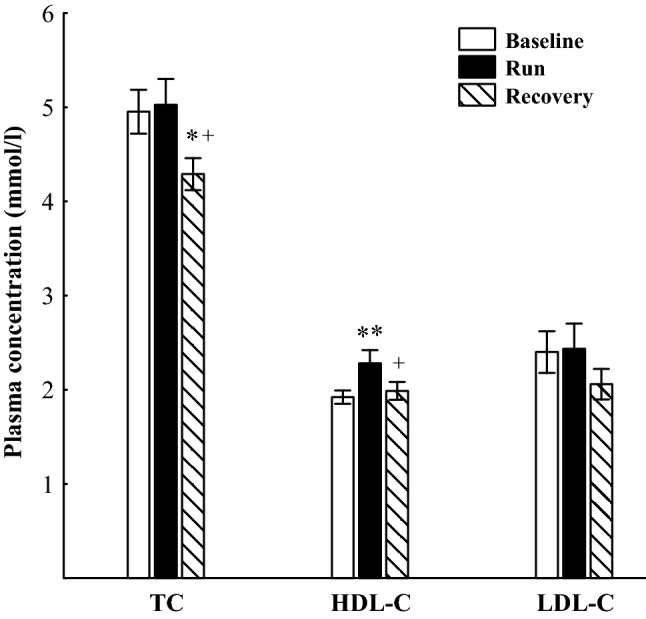
The plasma concentrations of total cholesterol (TC), high-density lipoprotein-cholesterol (HDL-C) and low-density lipoprotein-cholesterol (LDL-C) before (Baseline), immediately after ultra-marathon run (Run) and at 90 min of recovery (Recovery). The values are mean ± SEM (*n* = 10). Asterisks denote significant differences from the resting values: *p < 0.05, **p < 0.01. Crosses denote significant differences from the exercise values: ^+^*p* < 0.05.

**Fig. 3:**
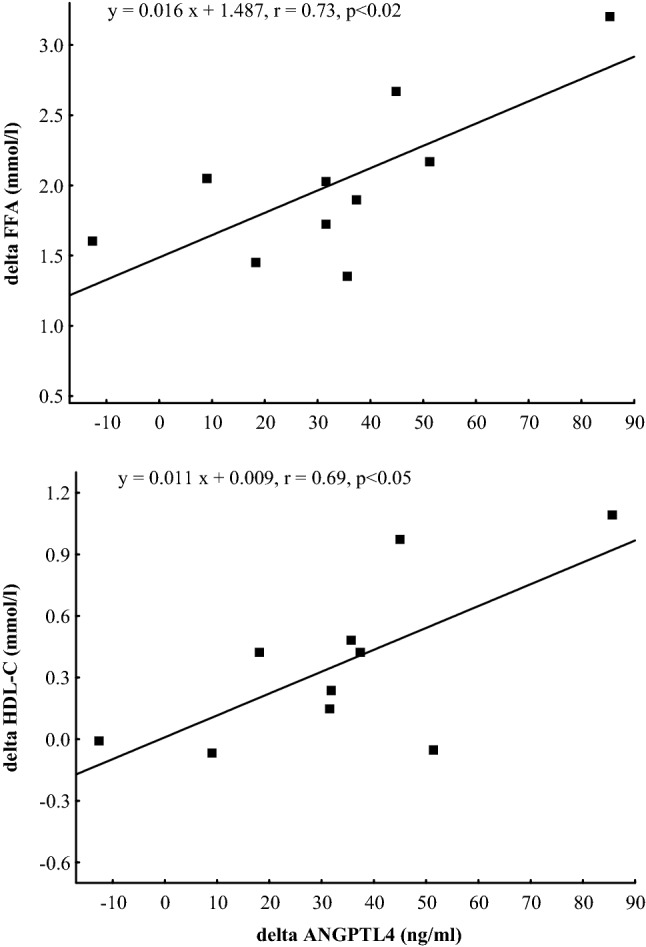
The relationships between exercise-induced changes in plasma angiopoietin-like protein 4 (ANGPTL4) and those of plasma free fatty acids (FFA) and high-density lipoprotein-cholesterol (HDL-C)

**Fig. 4:**
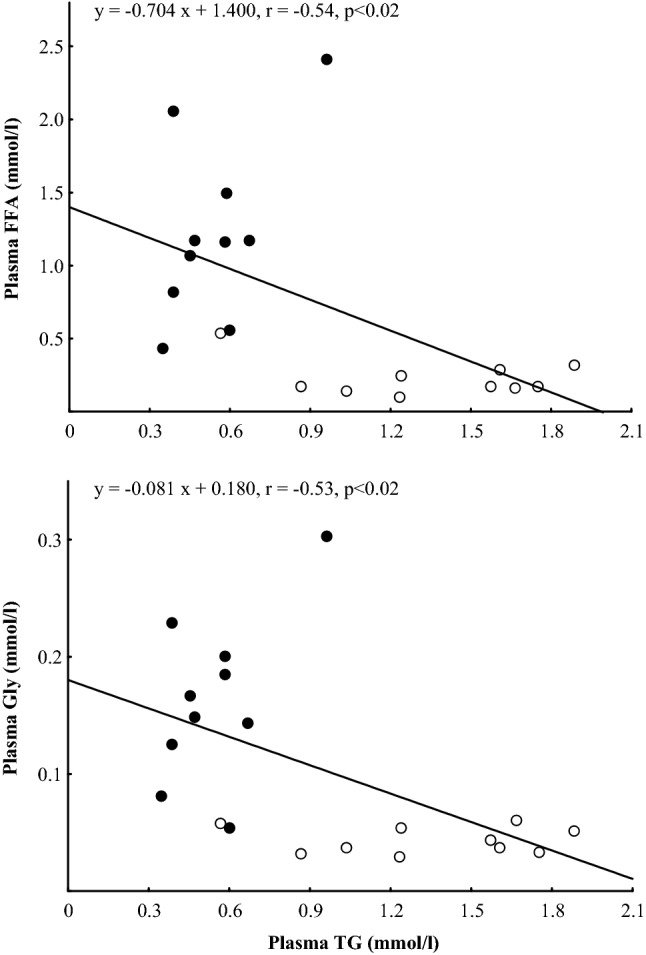
The relationships between the resting values of plasma triacylglycerols (TG), free fatty acids (FFA) and glycerol (Gly) concentrations before the run (open circle) and at 90 min of recovery (closed circle)

The ultra-marathon run caused significant increases in plasma concentrations of ANGPTL4 (*p* < 0.001), FFA and Gly (*p* < 0.01) as well as decreases in plasma TG (*p* < 0.01) (Fig. 1). Plasma HDL-C concentration immediately after the run was significantly increased (*p* < 0.01), whereas plasma TC and LDL-C did not differ from baseline (Fig. 2). After 90 min of recovery, plasma ANGPTL4 did not differ significantly from the exercise values and was still higher (*p* < 0.01) than before exercise. Plasma FFA and Gly were significantly lower than immediately after the run but still higher than at baseline (*p* < 0.01). Plasma TG did not differ from the exercise values and was significantly lower than before the run (*p* < 0.01). Plasma TC was significantly lower than immediately after and before the run (*p* < 0.05), whereas plasma HDL-C and LDL-C did not differ significantly from the baseline. The mean TC/HDL-C and TG/HDL-C molar ratios immediately after the run and after 90 min of recovery were significantly lower (*p* < 0.01) than at baseline (Table 2). The exercise-evoked changes in plasma ANGPTL4 concentration correlated positively with those of plasma FFA (*r* = 0.73, *p* < 0.02) and HDL-C (*r* = 0.69, *p* < 0.05) (Fig. 3). Significant positive correlation occurred between plasma concentrations of ANGPTL4 and FFA after 90 min of recovery (*r* = 0.77, *p* < 0.01). The negative relationships between plasma concentrations of TG and both FFA and Gly were found before the run and at 90 min of recovery period (*r* = − 0.54, *p* < 0.02; *r*  − 0.53, *p* < 0.02; respectively) (Fig. 4).

**Table 2:** The molar ratios of total cholesterol to high-density lipoprotein-cholesterol (TC/HDL-C) and triacylglycerols to high-density lipoprotein-cholesterol (TG/HDL-C) before (Baseline), immediately after ultra-marathon run (Run) and at 90 min of recovery (Recovery) (*n* = 10)

Parameters	Baseline	Run	Recovery
TC/HDL-C	2.61 ± 0.15	2.26 ± 0.16**	2.19 ± 0.11**
TG/HDL-C	0.71 ± 0.08	0.29 ± 0.03**	0.27 ± 0.02**

The values are means ± SEM. Asterisks denote significant differences from the resting values: **p < 0.01

No significant changes were seen in the haematocrit.

## Discussion

The new finding of the present study is that the 100-km mountain ultra-marathon run induced significant increase in the plasma ANGPTL4 concentration in endurance-trained young men. The rise in plasma ANGPTL4 was associated with increases in plasma FFA and Gly as well as decreases in plasma TG. No further increase in plasma ANGPTL4 concentration and a twofold reduction in plasma FFA were observed at 90 min of recovery. Furthermore, significant positive correlations were found between exercise-induced increases in plasma ANGPTL4 and those of plasma FFA as well as between plasma ANGPTL4 and FFA concentrations after 90 min of recovery.

The alterations in plasma lipids observed in this study might indicate a substantial mobilization of systemic fat stores and support the view that lipid oxidation is the predominant fuel source during submaximal exercise intensities (<65%* V*O_2max_) (Purdom et al. 2018). Comparable changes in lipid profile immediately after 24 h and 48 h ultra-marathons were observed by Waśkiewicz et al. (2012) and Kłapcińska et al. (2013). Helge et al. (2007) found that ultra-marathon run markedly increases the plasma fatty acids utilization to preserve blood glucose and muscle glycogen content. In a number of studies blood glucose concentration did not change during ultra-marathon races (Keul et al. 1981; Fallon et al. 1998; Waśkiewicz et al. 2012; Kłapcińska et al. 2013; Mrakic-Sposta et al. 2015). The pronounced fat oxidation observed by Helge et al. (2007) was associated with increases in LPL mRNA content, LPL activity, pyruvate dehydrogenase kinase 4 and forkhead homolog in rhabdomyosarcoma mRNA expression in skeletal muscle. The authors concluded that exercise-induced regulation of genes encoding proteins involved in fatty acid recruitment and oxidation may contribute to these changes. The time-dependent increases in fat oxidation and parallel reduction in oxidation of carbohydrates were observed also during cycling for 240 min at 57% of *V*O_2max_ (Watt et al. 2002). In other studies, a distinct rise in plasma FFA during 120–180 min cycling at 40–50% *V*O_2max_ was accompanied by elevated plasma ANGPTL4 (Kersten et al. 2009; Catoire et al. 2014). It was concluded that changes in plasma ANGPTL4 are mediated by elevated plasma FFA.

It is interesting to note that a tenfold increase in plasma FFA concentration, observed in the present study, occurred despite the fact that runners consumed carbohydrate-rich meals and drinks during the race.

Studies in mice and cultured myotubes (Norheim et al. 2014) showed that FFA and cortisol have the ability to increase skeletal muscle ANGPTL4 mRNA expression during exercise by activation of PPAR and glucocorticoid receptor, respectively. Some authors suggested that activation of PPAR-ANGPTL4 axis functions as a negative feedback mechanism that may serve to protect the muscle fibers from lipid overload, fatty acid-induced oxidative stress and lipid peroxidation (Georgiadi et al. 2010). Catoire et al. (2012, 2014) found that the stimulatory effect of plasma FFA on ANGPTL4 in exercising muscle is counteracted by AMPK-mediated suppression of ANGPTL4 mRNA. Increases in blood cortisol concentration were observed after both marathon and ultra-marathon races (Dessypris et al. 1975; Sundsfjord et al.^1975^; Keul et al. 1981). It was shown that cortisol may partially contribute to the adaptation of hepatic metabolism during exercise, which includes augmentation of gluconeogenesis and mobilization and use of fats for the production of energy. However, it should be noted that a major regulator of hepatic metabolism during exercise is a change in the glucagon-to-insulin ratio, which increases due to elevating glucagon and falling insulin level in the bloodstream (Weigert et al. 2019). Studies in humans demonstrated that this ratio is a regulator of plasma ANGPTL4 during exercise, probably involving glucagon-cAMP-PKA-driven hepatic ANGPT4 expression (Ingerslev et al. 2017). It was shown that liver and adipose tissue may contribute more than muscle to the exercise-induced increase in circulating ANGPTL4 (Norheim et al. 2014; Ingerslev et al. 2017).

Significant increases in plasma glucagon with concomitant decreases in plasma insulin concentration were observed after 65 km mountain ultra-marathon in trained runners (Sansoni et al. 2017). Decreases in insulin levels were also observed after 180 km ultra-marathon race in highly trained athletes (Roupas et al. 2013). It was demonstrated that insulin lowers plasma ANGPTL4 concentration in humans by lowering nonesterified fatty acids concentrations as well as by inhibiting ANGPTL4 expression and release. Insulin-mediated decrease in ANGPTL4 was attenuated by glucocorticoids (Kersten et al. 2009; Van Raalte et al. 2012).

In the present study, despite the increased plasma ANGPTL4, circulating TG levels were significantly decreased and there was no correlation between plasma ANGPTL4 and TG concentrations. This is in line with results obtained by other authors (Smart-Halajko et al. 2010; Robciuc et al. 2010, 2011; Tjeerdema et al. 2014), who also did not find any relationships between ANGTL4 and TG levels. It seems likely that the exercise-induced decreases in plasma TG may result from LPL-mediated hydrolysis of TG-VLDL and TG-chylomicrons. Gill and Hardman (2003) believe that the energy expended during exercise is an important determinant of exercise-induced TG reduction and that both increased LPL-mediated hydrolysis of TG and reduced hepatic TG-VLDL secretion contribute to the plasma TG lowering effect of exercise. It seems likely that this effect results from the AMPK–mediated down regulation of ANGPTL4 mRNA and hence enhanced LPL activity in working muscle, which promotes the use of circulating TG as fuel for active muscles (Catoire et al. 2014). The exercise-induced TG-lowering can also result from the glycosylphosphatidylinositol-anchored HDL-binding protein 1 (GPIHBP1)-mediated down regulation of ANGPTL4. Sonnenburg et al. (2009) demonstrated that GPIHBP1 stabilizes LPL and plays an important role in buffering the inactivation of LPL by ANGPTL4. On the other hand, Chi et al. (2015) observed that ANGPTL4 can bind and inactivate LPL complexed to GPIHBP1 on the surface of endothelial cells and that inactivation of LPL by ANGPTL4 reduces the affinity of LPL for GPIHBP1. Thus, the role of ANGPTL4 in regulating TG metabolism remains unclear.

The present study also showed significant increases in plasma Gly immediately after exercise as well as at 90 min of recovery. It is well known that plasma Gly concentration, similarly to plasma FFA, is a good indicator of adipose lipolysis during aerobic exercise (Lewis et al. 2010). The significant inverse correlations between the plasma concentrations of TG and both FFA and Gly may indicate that a fraction of circulating FFA and Gly originates from the hydrolysis of TG-rich lipoproteins. There are many evidences that ANGPTL4 may independently from catecholamines and other factors stimulates adipocyte tissue lipolysis, resulting in increased release of FFA into the circulation (Robciuc et al. 2011; Gray et al. 2012; McQueen et al. 2017). McQueen et al. (2017) showed that C-terminal fibrinogen-like domain of ANGPTL4 participates in this activity. Significant positive correlation between the plasma concentrations of ANGPTL4 and FFA found in the present study may indicate that elevated level of ANGPTL4 increases plasma FFA concentration and increased concentration of FFA would increase release of ANGPTL4.

Additional finding of the present investigation is that the ultra-marathon run caused significant increases in plasma HDL-C without significant changes in plasma TC and that the exercise-induced increases in plasma ANGPTL4 correlated positively with those of plasma HDL-C. Similar changes in plasma TG and TC concentrations as well as in TG/HDL-C and TC/HDL-C molar ratios were observed in ultra-endurance runners who completed a 24-h or 48-h ultra-marathon race (Waśkiewicz et al. 2012; Kłapcińska et al. 2013). Emed et al. (2016) reported a trend in reduction of TG and TC, without significant changes in plasma HDL-C and LDL-C levels in male athletes that participated in 133 km ultra-marathon race. In Wu et al. studies (2004) plasma TC and HDL-C concentrations remained unchanged immediately after 100 km ultra-marathon race, but it was significantly decreased on the second and ninth days after the race.

The data concerning the relationship between circulating ANGPTL4 and cholesterol are controversial. Some authors (Stejskal et al. 2008; Smart-Halajko et al. 2010; Tjeerdema et al. 2014) found inverse correlations between plasma concentrations of ANGPTL4 and HDL-C or TC, while others did not find such relationships in healthy subjects (Robciuc et al. 2010; Yang et al. 2017). Studies by Mandard et al. (2006) and Yang et al. (2017) showed that ANGPTL4 is present in HDL particles and physically protects HDL from endothelial lipase-mediated hydrolysis as well as sustains HDL function. Thus, the increase in plasma HDL-C concentration immediately after the run and positive correlation between the exercise-induced changes in plasma ANGPTL4 and those of plasma HDL-C observed in the present study might support the hypothesis that the main lipoprotein parameter related to ANGPTL4 is HDL-C, not TG (Robciuc et al. 2011).

## Limitations of the study

A limitation of this study is that we did not measure glucose, cortisol, glucagon and insulin blood levels in the runners, while changes in these variables may play a role in ANGPTL4 response to exercise. Further research is required to elucidate the role of these important factors in regulation of ANGPTL4 during ultra-marathon race.

## Conclusions

In summarizing, the present study demonstrated that the 100-km mountain ultra-marathon running induced significant increase in the plasma ANGPTL4 concentration, which is likely mediated by elevated plasma FFA. These results suggest that increase in ANGPTL4 secretion may be a compensatory mechanism against fatty acid-induced oxidative stress. Increase in plasma HDL-C observed immediately after the run can be due to the protective effect of ANGPTL4 on HDL. Inverse correlations between the plasma TG and both FFA and Gly concentrations may indicate that a fraction of circulating FFA and Gly originates from the hydrolysis of TG-rich lipoproteins. Decreases in TG/HDL-C and TC/HDL-C ratios prove the beneficial effect of physical activity on lipid profiles.

## Abbreviations

ANGPTL4: Angiopoietin like protein 4
FFA: Free fatty acids
Gly: Glycerol
HDL: High-density lipoprotein
HDL-C: High-density lipoprotein-cholesterol
LPL: Lipoprotein lipase
LDL-C: Low-density lipoprotein-cholesterol
TC: Total cholesterol
TG: Triacylglycerols
VLDL: Very low-density lipoproteins
*V*O_2max_: Maximal oxygen uptake
